# Rottlerin exerts its anti-tumor activity through inhibition of Skp2 in breast cancer cells

**DOI:** 10.18632/oncotarget.11614

**Published:** 2016-08-25

**Authors:** Xuyuan Yin, Yu Zhang, Jingna Su, Yingying Hou, Lixia Wang, Xiantao Ye, Zhe Zhao, Xiuxia Zhou, Yali Li, Zhiwei Wang

**Affiliations:** ^1^ The Cyrus Tang Hematology Center and Collaborative Innovation Center of Hematology, Jiangsu Institute of Hematology, The First Affiliated Hospital, Soochow University, Suzhou, China; ^2^ Department of Oncology, Guizhou People's Hospital, Guizhou, China; ^3^ Department of Anesthesiology, Shenzhen People's Hospital, Shenzhen Anesthesiology Engineering Center, The Second Clinical Medical College, Jinan University, Guangdong, China; ^4^ Department of Pathology, Beth Israel Deaconess Medical Center, Harvard Medical School, Cambridge, MA, USA

**Keywords:** rottlerin, breast cancer, Skp2, invasion, apoptosis

## Abstract

Studies have investigated the tumor suppressive role of rottlerin in carcinogenesis. However, the molecular mechanisms of rottlerin-induced anti-tumor activity are largely unclear. Skp2 (S-phase kinase associated protein 2) has been validated to play an oncogenic role in a variety of human malignancies. Therefore, inactivation of Skp2 could be helpful for the treatment of human cancers. In the current study, we explore whether rottlerin could inhibit Skp2 expression, leading to inhibition of cell growth, migration and invasion in breast cancer cells. We found that rottlerin treatment inhibited cell growth, induced apoptosis and cell cycle arrest. We also revealed that rottlerin suppressed cell migration and invasion in breast cancer cells. Mechanically, we observed that rottlerin significantly down-regulated the expression of Skp2 in breast cancer cells. Importantly, overexpression of Skp2 abrogated rottlerin-mediated tumor suppressive activity, whereas down-regulation of Skp2 enhanced rottlerin-triggered anti-tumor function. Strikingly, we identified that rottlerin exhibited its anti-tumor potential partly through inactivation of Skp2 in breast cancer. Our findings indicate that rottlerin could be a potential safe agent for the treatment of breast cancer.

## INTRODUCTION

Breast cancer is one of the common diagnosed malignancies and the main cause of cancer mortality among females worldwide [1]. In the United States, approximately 246,660 new breast cancer cases were occurred in female in 2016, and 40,450 Americans will die from this disease [2]. Some signaling pathways have been identified to play an oncogenic role in the development and progression of breast cancer. For example, Akt, mTOR (mammalian target of rapamycin), MAPK (mitogen-activated protein kinases), NF-κB (nuclear factor-κB), Notch, SHH (sonic hedgehog) pathways have been validated to be positively associated with breast tumorigenesis [3–6]. Recently, Skp2 (S-phase kinase associated protein 2), which belongs to the ubiquitin proteasome system (UPS), is the substrate-recruiting component of the SCF (Skp1-Cullin1-F-box complex) type of E3 ubiquitin ligase complex [7]. Skp2 functions as an oncoprotein in a variety of human cancers [8, 9]. Skp2 exerts its oncogenic function through targeting its substrates [10]. Skp2 has been revealed to critically enhance the pathogenesis of breast cancer [3]. Due to its oncogenic role in tumorigenesis, inhibition of Skp2 could be a promising therapeutic strategy for combating breast cancer.

Recently, rottlerin, also known as mallotoxin, has been extensively investigated and identified as an inhibitor of PKCδ (protein kinase C δ) [11]. PKCδ could enhance tumorigenesis in multiple human cancers [12]. Rottlerin could indirectly inhibit the activity of PKCδ. Moreover, rottlerin could inhibit some other protein kinases including Akt/PKB (protein kinase B), PRAK (p38-regulated/activated protein kinase), MAPKAP-2 (mitogen-activated protein kinase-activated protein kinase 2), and CaMK (calcium/calmodulin-dependent protein kinase) [11]. Furthermore, rottlerin sensitized tumor cells to TRAIL (tumor necrosis factor-related apoptosis-inducing ligand)-triggered apoptosis through regulation of mitochondrial function independent of PKCδ in colon carcinoma [13]. Similarly, rottlerin sensitized glioma cells to TRAIL-mediated apoptosis via down-regulation of Cdc2 (cell cycle division 2) and subsequent suppression of XIAP (X-chromosome-linked IAP) and survivin [14]. Rottlerin inhibited the NF-κB and cyclin D1 in MCF-7 breast cancer cells, leading to cell proliferation inhibition [15]. Controversially, one study showed that rottlerin enhanced oncoprotein COX-2 (cyclooxygenase-2) expression via sustained p38 MAPK (mitogen-activated protein kinase) activation in breast cancer cells [16], suggesting that further investigation is required to determine the function of rottlerin in tumorigenesis.

In the current study, we investigated the physiological function of rottlerin on cell growth, apoptosis, cell cycle, migration, and invasion in breast cancer cells. Moreover, we explored the molecular mechanism of rottlerin-mediated anti-tumor activity in breast cancer cells. Our findings demonstrated that rottlerin inhibited cell growth, triggered apoptosis, induced G0/G1 cell cycle arrest, and suppressed cell migration and invasion. More importantly, we identified that rottlerin-induced anti-cancer activity is partly through down-regulation of Skp2 pathway in breast cancer. Our data indicates that rottlerin could be a potential efficient agent for the treatment of breast cancer.

## RESULTS

### Rottlerin inhibited cell proliferation

**Figure 1:**
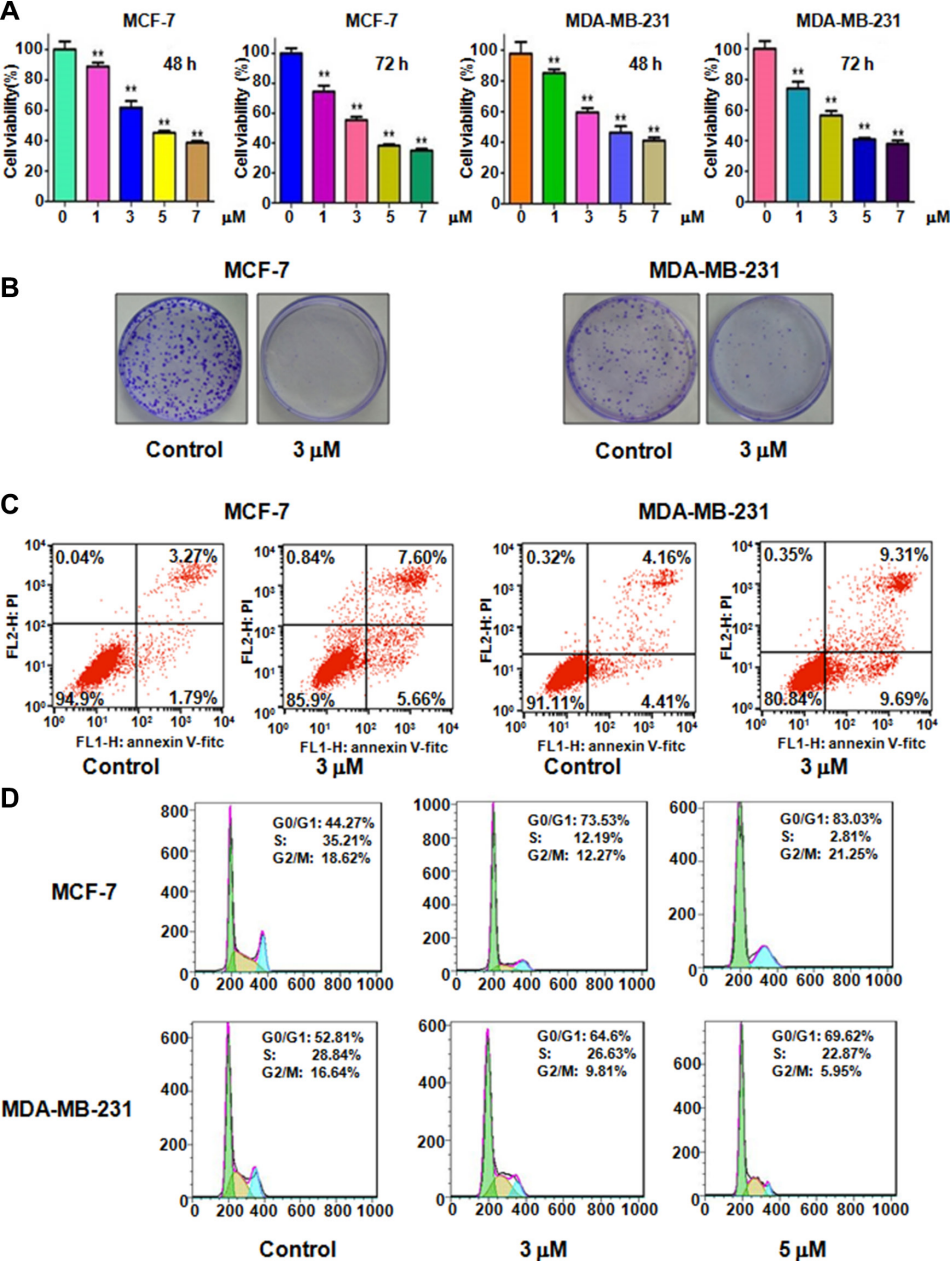
Effect of rottlerin on cell growth, apoptosis, and cell cycle (**A**) CellTiter-Glo^®^ luminescence assay was performed to detect the cell growth in breast cancer cells treated with different concentrations of rottlerin. *P < 0.05, compared to the control (DMSO treatment). (**B**) Colony formation analysis was conducted to measure the colony numbers in breast cancer cells treated with rottlerin. (**C**) Flow cytometry was used to detect the cell apoptosis in breast cancer cells treated with rottlerin. (**D**) Cell cycle was analyzed by Flow cytometry in rottlerin-treated breast cancer cells.

To determine whether rottlerin suppresses cell proliferation, we performed CTG assay in MCF-7 and MDA-MB-231 cells after different concentrations of rottlerin treatment for 48 hours and 72 hours, respectively. As demonstrated in Figure 1A, rottlerin significantly inhibited cell proliferation in dose-dependent manner. Specifically, 3 μM and 5 μM rottlerin treatments in both MCF-7 and MDA-MB-231 cells led to 40% and 60% cell proliferation inhibition at 72 hours, respectively. Thus, we conducted the following studies using 3 μM and 5 μM rottlerin. Moreover, the results from colony forming assay showed that treatment of breast cancer cells with 3 μM rottlerin remarkably reduced the colony numbers (Figure 1B). These data indicated that rottelrin inhibited cell proliferation in both breast cancer cells.

### Rottlerin induced cell apoptosis

Previous studies have shown that rottlerin triggered apoptosis in breast cancer cells [17, 18]. We next aim to further validate whether cell proliferation inhibition by rottlerin treatment could be due to rottlerin-mediated cell apoptotic death. To this end, PI-FITC-annexin assay was performed to detect the cell apoptosis in both MCF-7 and MDA-MB-231 cells treated with 3 μM Rottlerin for 48 hours. We observed that rottlerin significantly induced cell apoptosis in both breast cancer cells (Figure 1C). Rottlerin treatment led to apoptotic cells from 5% to 13% in MCF-7 cells, and from 8.5% to 19% in MDA-MB-231 cells (Figure 1C). Consistent with other studies [17, 18], rottlerin could stimulate apoptosis in breast cancer cells.

### Rottlerin induced cell cycle arrest

Next, we explored whether cell cycle arrest contributed to cell growth inhibition in breast cancer cells after rottlerin treatment. Cell cycle analysis by PI staining and flow cytometry in MCF-7 and MDA-MB-231 cells treated with different concentrations of rottlerin. Our results demonstrated that rottlerin treatment caused cell cycle arrest at G1 phase. Specifically, 3 μM and 5 μM rottlerin led to G1 cell population from 44% to 73.5% to 83%, respectively, in MCF-7 cells (Figure 1D). Similar result was found in MDA-MB-231 cells (Figure 1D). Our observations implied that rottlerin could induce G1 cell cycle arrest.

### Rottlerin inhibited cell migration and invasion

**Figure 2:**
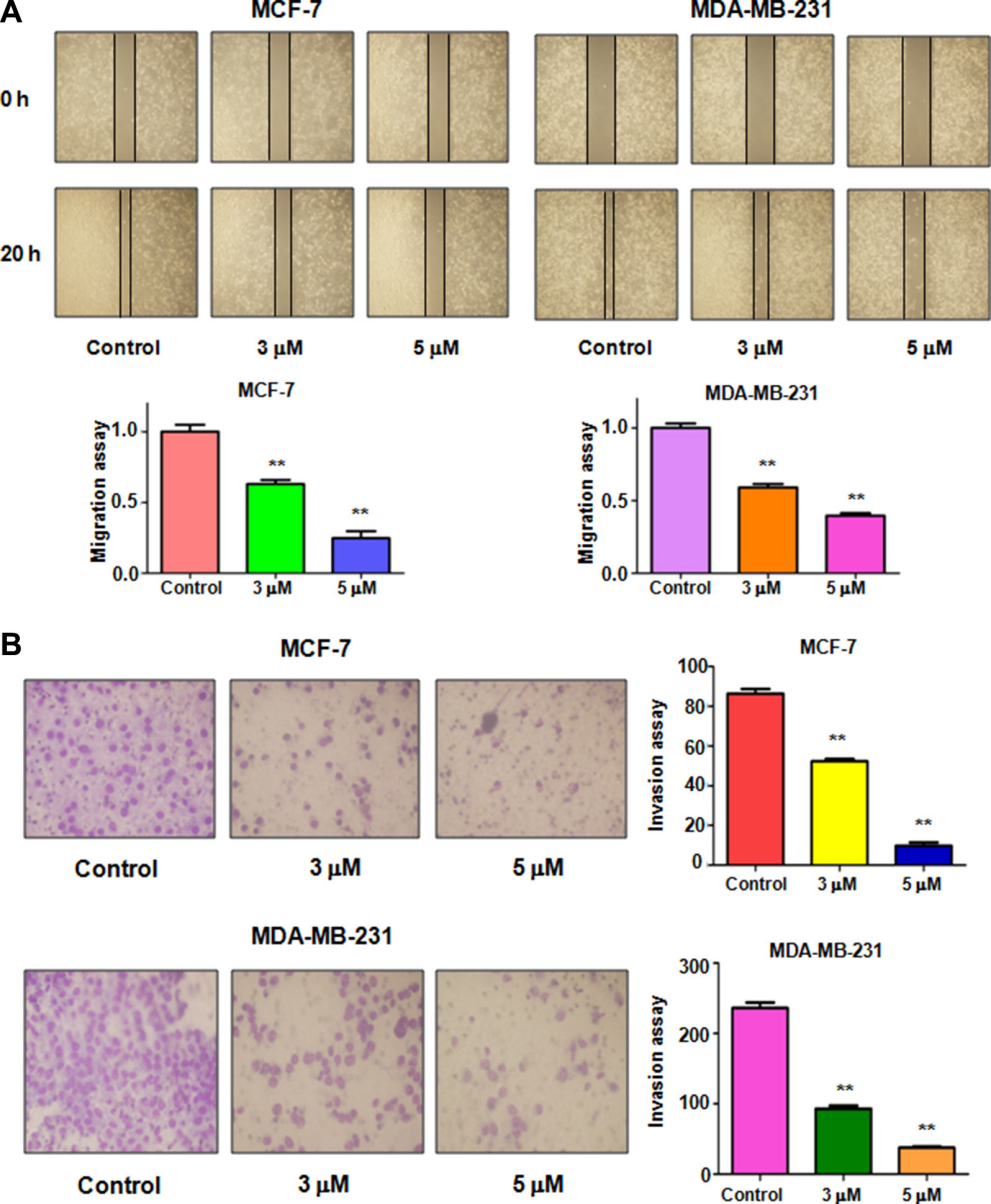
Effect of rottlerin on cell migration and invasion (**A**) Top panel: Wound healing assay was performed to detect the inhibitory effect of rottlerin on MCF-7 cells and MDA-MB-231 cells after rottlerin treatment. Bottom panel: Quantitative results are illustrated for left panel. **P < 0.01 vs control (DMSO treatment). (**B**) Left panel: Transwell chambers assay was conducted to measure the cell invasion in MCF-7 cells and MDA-MB-231 cells after rottlerin treatment. Right panel: Quantitative results are illustrated for left panel. **P < 0.01 vs control.

To determine whether rottlerin inhibited cell migratory activity, the wound healing assay was conducted in MCF-7 and MDA-MB-231 cells treated with rottlerin. We found that rottlerin treatment remarkably suppressed cell migration in dose-dependent manner in both breast cancer cells (Figure 2A). To further define whether rottlerin has invasion inhibition potential, invasion assay was applied for measuring penetration of breast cancer cells via the matrigel-coated membrane. Our Transwell invasion assay revealed that rottlerin decreased the cell numbers of penetration through matrigel, suggesting that rottlerin could retard the cell invasion in breast cancer cells (Figure 2B).

### Rottlerin decreased Skp2 expression

**Figure 3:**
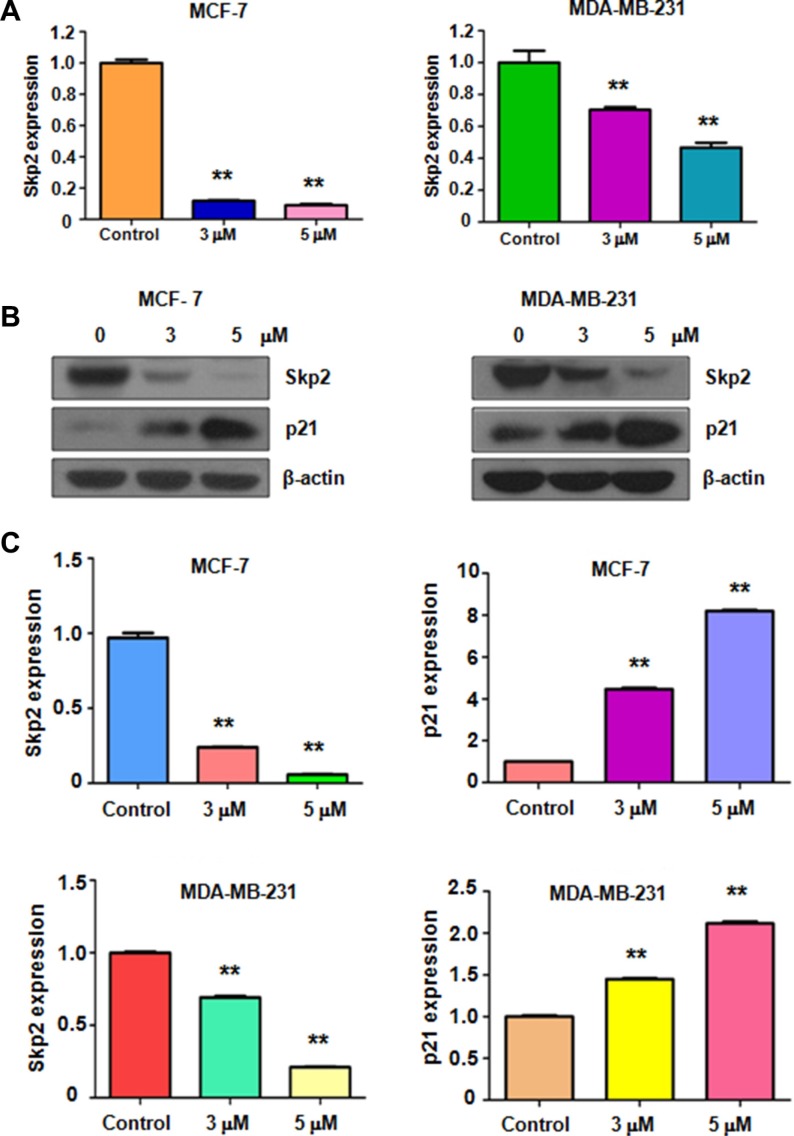
Rottlerin inhibited Skp2 expression at RNA and protein levels (**A**) RT-PCR was used to detect the Skp2 mRNA expression in breast cancer cells treated with rottlerin. **P < 0.01 vs control (DMSO treatment). (**B**) Western blotting analysis was conducted to determine the expression of Skp2 and p21 in MCF-7 and MDA-MB-231 cells after rottlerin treatment. (**C**) Quantitative results are illustrated for panel B. **P < 0.01 vs control.

Skp2 has been characterized as an oncoprotein in breast cancer. Therefore, inhibition of Skp2 could be a promising approach for treating breast cancer. Next, we explored whether rottlerin could down-regulate Skp2 expression, leading to its anti-tumor activity in breast cancer cells. Real-time PCR and Western blotting were used to detect the expression of Skp2 at mRNA and protein levels, respectively, in breast cancer cells treated with rottlerin. The results showed that both Skp2 mRNA and its protein level were significantly decreased in rottlerin-treated cells (Figure 3A, 3B). We also found that the expression of p21, one of Skp2 downstream target, was upregulated in cells after rottlerin treatment (Figure 3B and 3C). Our data validate that rottlerin inhibited Skp2 expression in breast cancer cells, indicating that rottlerin could function as a Skp2 inhibitor.

### Over-expression of Skp2 abrogated rottlerin-mediated anti-tumor activities

**Figure 4:**
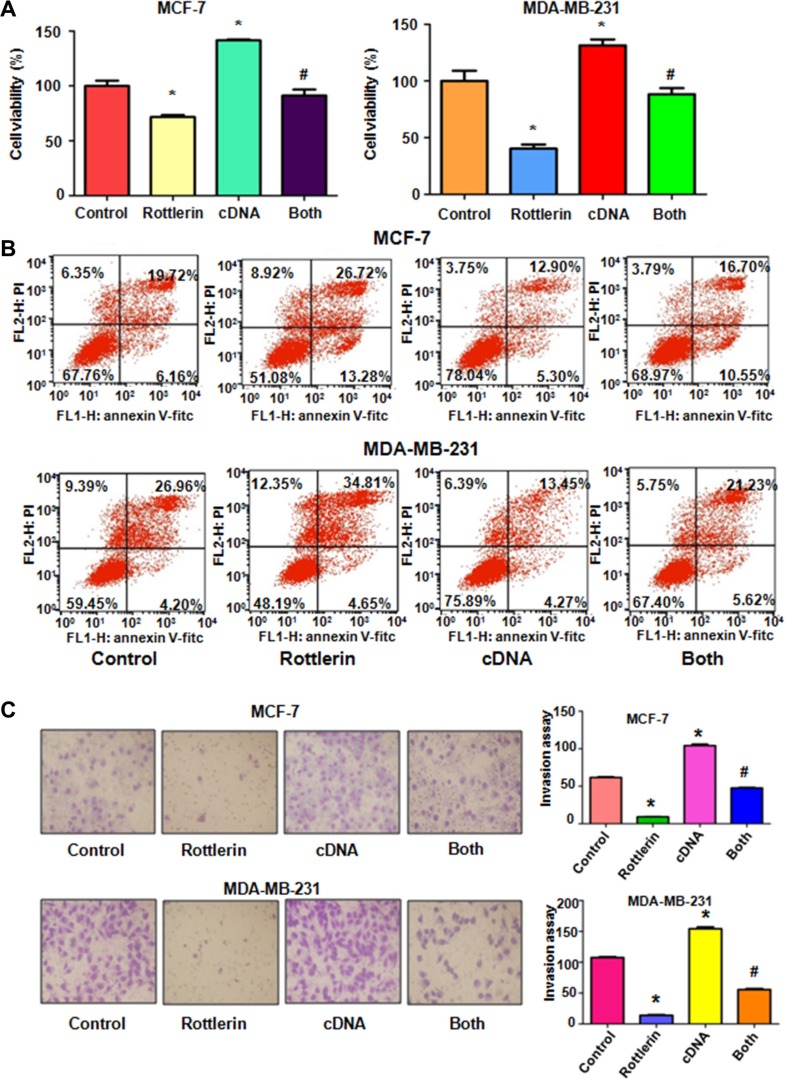
The effect of Skp2 overexpression on cell growth, apoptosis and invasion (**A**) CellTiter-Glo^®^ luminescence assay was used to investigate the effect of Skp2 overexpression in combination with rottlerin treatment on breast cancer cell growth. Control: pcDNA3.1. cDNA: Skp2 cDNA; Both: Skp2 cDNA+rottlerin. *P < 0.05, compared with control; ^#^*P* < 0.05 compared with rottlerin treatment alone or Skp2 cDNA transfection alone. (**B**) FACS was conducted to detect cell apoptosis in breast cancer cells after Skp2 cDNA transfection and rottlerin treatment. (**C**) Left panel, Invasion assay was performed in MCF-7 and MDA-MB-231 cells after Skp2 cDNA transfection and rottlerin treatment. Right panel, Quantitative results are illustrated for left panel. *P < 0.05.

**Figure 5:**
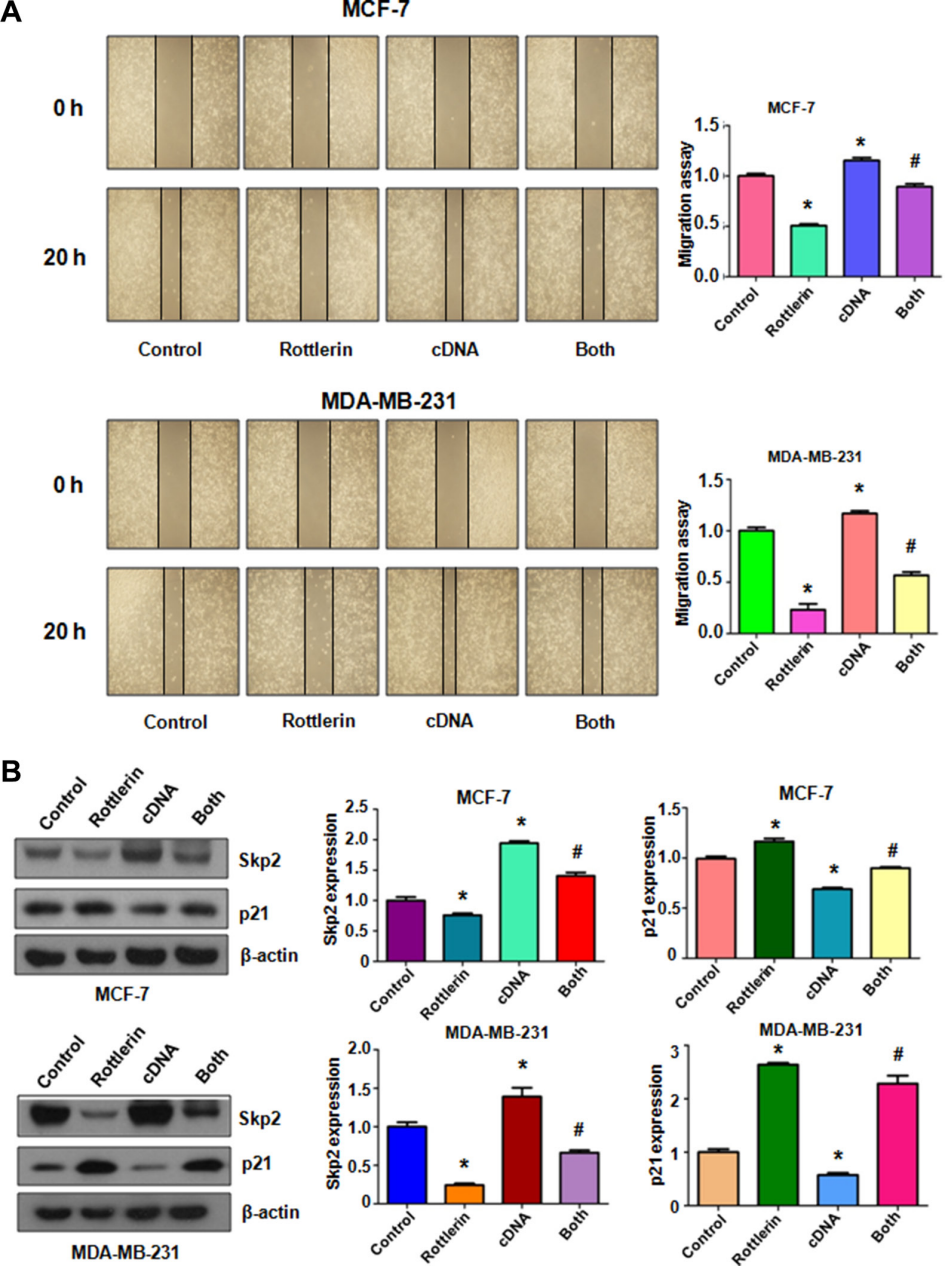
The effect of Skp2 overexpression on cell migration (**A**) Left panel: The wound healing assay was conducted to test the cell migration after Skp2 cDNA transfection and rottlerin treatment. Control: pcDNA3.1. cDNA: Skp2 cDNA; Both: Skp2 cDNA+rottlerin. Right panel, Quantitative results are illustrated for left panel. *P < 0.05, compared with control; ^#^*P* < 0.05 compared with rottlerin treatment alone or Skp2 cDNA transfection alone. (**B**) Left panel, the expression of Skp2 and its target p21 was measured by Western blotting in breast cancer cells with Skp2 cDNA transfection and rottlerin treatment. Right panel, Quantitative results are illustrated for left panel. *P < 0.05, compared with control; ^#^*P* < 0.05 compared with rottlerin treatment alone or Skp2 cDNA transfection alone.

To further explore whether rottlerin exerts its anti-cancer activity via inhibition of Skp2 in breast cancer cells, Skp2 cDNA was transfected into MCF-7 and MDA-MB-231 cells to upregulate Skp2 expression. The Skp2-overexpressing cells were treated with rottlerin for 48 hours. We found that overexpression of Skp2 enhanced cell proliferation in both breast cancer cell lines (Figure 4A). Importantly, up-regulation of Skp2 rescued cell proliferation inhibition by rottlerin treatment in breast cancer cells (Figure 4A). Consistently, overexpression of Skp2 by its cDNA transfection inhibited cell apoptosis in breast cancer cells (Figure 4B). Moreover, up-regulation of Skp2 abrogated rottlerin-induced apoptosis in both breast cancer cells (Figure 4B). Notably, Skp2 overexpression led to enhanced cell invasion in breast cancer cells (Figure 4C). Additionally, overexpression of Skp2 in combination with rottlerin resulted in more numbers of invasive cells compared with rottlerin treatment alone (Figure 4C). In line with this, we observed the similar results of migration by wound healing assay in Skp2-overexpressing cells treated with rottlerin (Figure 5A). Skp2 overexpression promoted cell migration and abrogated rottlerin-inhibited cell migratory activity (Figure 5A). Mechanistically, we found that Skp2 cDNA transfection significantly increased the Skp2 expression and abrogated rottlerin-mediated Skp2 inhibition (Figure 5B). We also observed that overexpression of Skp2 abrogated the inhibition of pAkt by rottlerin (Supplementary Figure S1). Altogether, rottlerin exerts its tumor suppressive function partly through down-regulation of Skp2 in breast cancer cells.

### Down-regulation of Skp2 by its siRNA promoted rottlerin-induced anti-tumor activity

**Figure 6:**
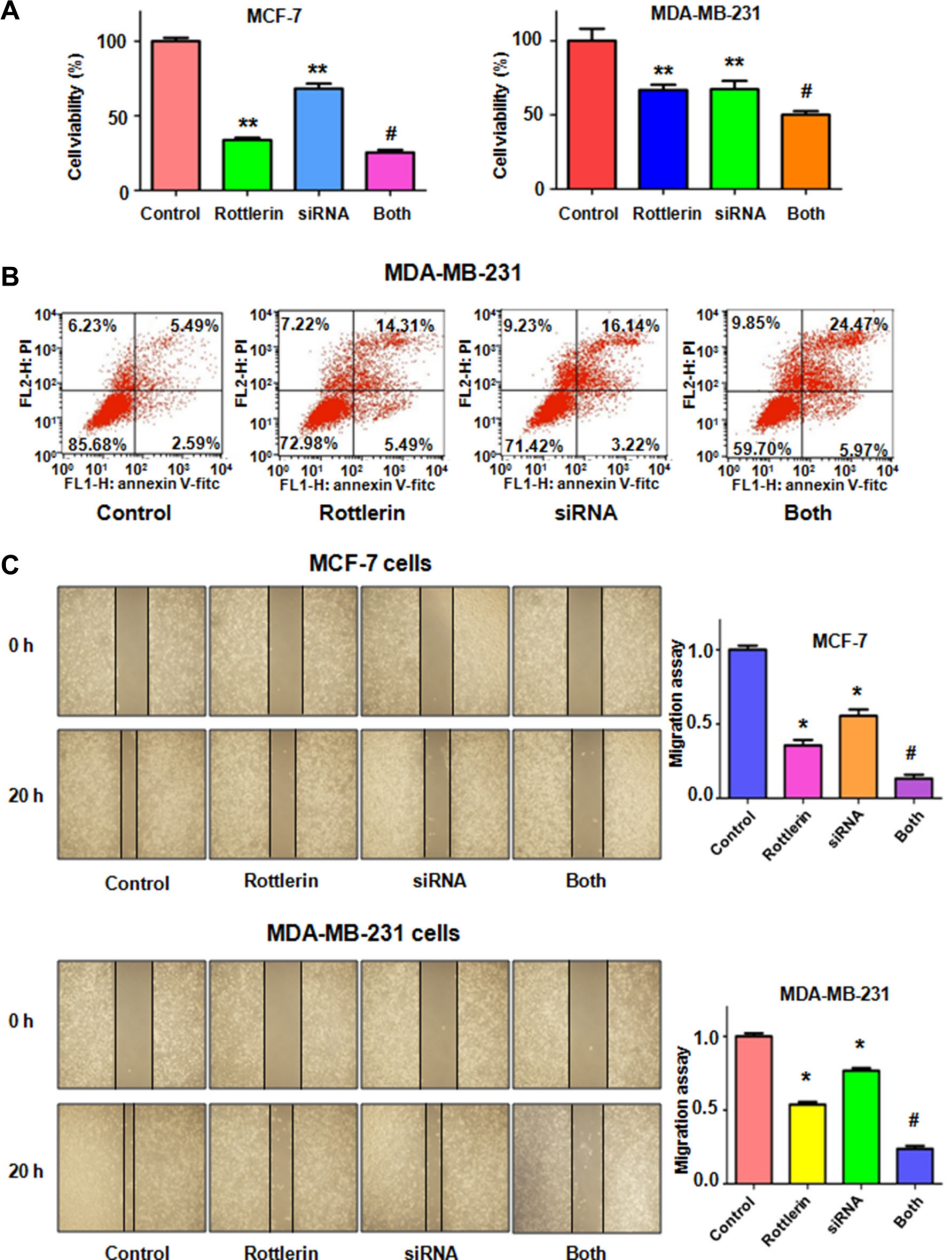
The effect of Skp2 downregulation on cell growth, apoptosis and migration (**A**) CellTiter-Glo^®^ luminescence assay was applied for detecting the effect of skp2 siRNA in combination with rottlerin treatment on breast cancer cell growth. *P < 0.05, compared with control; ^#^*P* < 0.05 compared with rottlerin treatment or Skp2 siRNA transfection. Control: siRNA control; SiRNA: Skp2 siRNA; Both: rottlerin + Skp2 siRNA. (**B**) Apoptosis was determined by Flow cytometry in MDA-MB-231 with Skp2 siRNA transfection and rottlerin treatment. (**C**) Left panel: The wound healing assay was used to investigate the cell migration in breast cancer cells after Skp2 siRNA transfection and rottlerin treatment. Right panel, Quantitative results are illustrated for left panel. *P < 0.05, compared with control; ^#^*P* < 0.05 compared with rottlerin treatment or Skp2 siRNA transfection.

**Figure 7:**
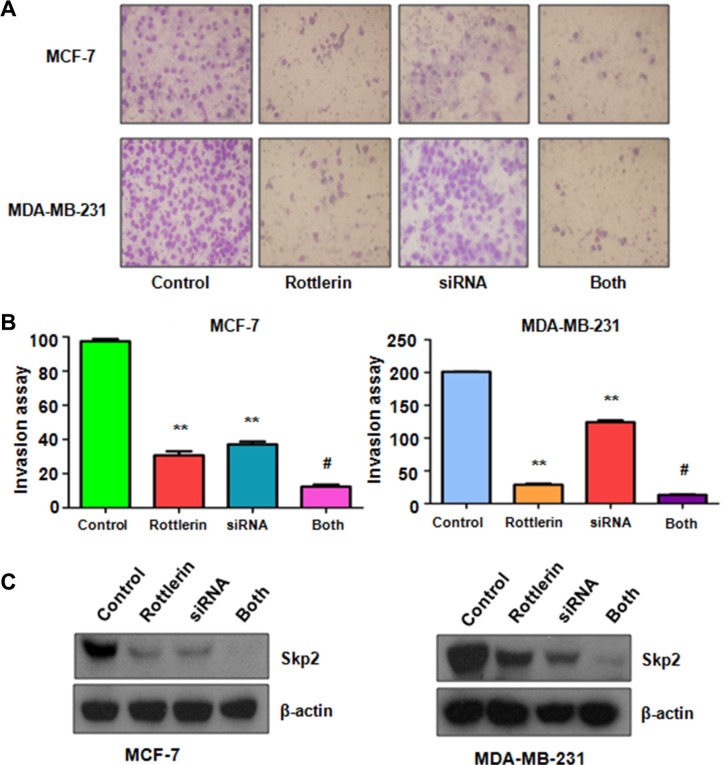
The effect of Skp2 downregulation on cell invasion (**A**) Invasion assay was performed in breast cancer cells after Skp2 siRNA transfection and rottlerin treatment. Control: siRNA control; SiRNA: Skp2 siRNA; Both: rottlerin + Skp2 siRNA. (**B**) Quantitative results are illustrated for panel A. *P < 0.05, vs control; ^#^*P* < 0.05 vs rottlerin treatment or Skp2 siRNA transfection. (**C**) The expression of Skp2 was tested by Western blotting analysis in breast cancer cells with Skp2 siRNA transfection and rottlerin treatment.

To further determine the oncogenic role of Skp2 in rottlerin-triggered anti-tumor function, Skp2 was depleted by its siRNA transfection in breast cancer cells treated with rottlerin. We observed that depletion of Skp2 suppressed cell growth in MCF-7 and MDA-MB-231 cells (Figure 6A). Cells with Skp2 siRNA transfection were more sensitive to rottlerin-mediated cell growth inhibition (Figure 6A). Consistently, depletion of Skp2 led to more apoptotic cells induced by rottlerin compared with rottlerin alone or Skp2 transfection alone (Figure 6B). Moreover, the results from wound healing assay showed that down-regulation of Skp2 inhibited cell migration (Figure 6C) and invasion (Figure 7A, 7B). Rottlerin treatment in combination with Skp2 depletion caused inhibition of cell migration and invasion to a greater degree (Figures 6C and 7A, 7B). Our results indicated that rottlerin could inhibit Skp2 expression, leading to its anti-tumor function in breast cancer cells. Notably, we found that Skp2 siRNA plus rottlerin inhibited Skp2 expression to more degree compared to rottlerin alone or siRNA transfection alone (Figure 7C).

## DISCUSSION

Accumulated evidence has revealed that Skp2 plays an oncogenic role in tumorigenesis [19, 20]. It is clear that Skp2 exerts its physiological function via degradation of its substrates including p21 [21], p27 [22], p57 [23], p53 [24], and Foxo1 [25]. Overexpression of Skp2 is associated with poor prognosis in breast cancer [23, 26]. Moreover, overexpression of Skp2 promoted cell proliferation in breast cancer cells [26], whereas inhibition of Skp2 suppressed cell proliferation in MDA-MB-231 cells [27]. In line with this, we observed the similar results, suggesting that Skp2 contributes to breast cancer cell growth. Hung et al. reported that overexpression of Skp2 enhanced cell invasion in lung cancer cells [28]. In the current study, we observed that up-regulation of Skp2 promoted cell migration and invasion, while down-regulation of Skp2 retarded cell migratory activity in breast cancer cells. Our findings demonstrated that Skp2 is an oncoprotein in breast cancer and targeting Skp2 could be a useful approach for the treatment of breast cancer.

Recently, multiple studies have demonstrated that rottlerin exhibited its anti-tumor activity in human cancers. For instance, rottlerin induced LRP6 degradation and inhibited mTORC1 and Wnt/β-catenin signaling in breast cancer cells [29]. Rottlerin inhibited the activation of caspase-2 via ubiquitin proteasome-mediated pathway, resulting in cell apoptosis in HeLa cells and ovarian cancer cells [30]. One study identified that rottlerin stimulated cell apoptosis via interactions with proteins of the Bcl-2 family in pancreatic cancer cells [31]. Remarkably, rottlerin inhibited pancreatic cancer cell growth via targeting multiple signaling pathways including Akt, Notch, and Shh (sonic hedgehog) pathways [32]. Interestingly, Rottlerin was found to induce autophagy in several cancer cell lines [33]. Rottlerin-mediated autophagy is through a PKCδ-independent manner in human fibrosarcoma cells [34]. Similarly, rottlerin triggered autophagy via suppression of PI3K/Akt/mTOR pathway in prostate [35], breast [17], and pancreatic cancer cells [36]. Rottlerin retarded migration in follicular thyroid carcinoma cells through destabilization of the focal adhesion complex [37]. In support of these findings, we found that rottlerin inhibited cell growth, induced apoptosis, arrested cell cycle, and retarded migration and invasion in breast cancer.

Due to the oncogenic function of Skp2 in tumorigenesis, inactivation of Skp2 could be helpful for treating human cancers. To this end, multiple groups have discovered several Skp2 inhibitors. For example, Compound A has been found to block Skp2 E3 ligase activity [38]. Another Skp2 inhibitor, namely Compound 25, also named as SZL-P1-41, was reported to inhibit Akt-mediated glycolysis and induced cellular senescence [39]. Since these chemical inhibitors exhibit side effects, it is important to discover natural agents with non-toxic nature to inactivate Skp2 in human cancer. In fact, several natural compounds have been reported including curcumin [40], butylidenephthalide [41], Flavokawain A (FKA) [42], and Salinomycin [43]. In the present study, we explored whether a nature agent rottlerin could be a potential inhibitor of Skp2 in breast cancer. We discovered that rottlerin inhibited Skp2 expression in breast cancer cells. More importantly, we confirmed that rottlerin exerts its anti-tumor function via inactivation of Skp2. Taken together, inhibition of Skp2 by rottlerin could be a promising approach for breast cancer treatment. However, more investigations are necessary to determine the functions of rottlerin in animal model and clinical trial in the future.

## MATERIALS AND METHODS

### Cell culture and reagents

Human MCF-7 and MDA-MB-231 cells were cultured in DMEM medium supplemented with 10% fetal bovine serum and 1% penicillin/streptomycin in a 5% CO_2_ atmosphere at 37°C. Primary antibody for Skp2 (SC-7164) was purchased from Santa Cruz Biotechnology (Santa Cruz, CA). Anti-p21 antibody was brought from Cell Signaling Technology. All secondary antibodies were purchased from Thermo Scientific. Lipofectamine 2000 was purchased from Invitrogen. Monoclonal anti-β-actin antibody and rottlerin (CAS number 82-08-6, 85% rottlerin) were obtained from Sigma-Aldrich (St. Louis, MO). Rottlerin was dissolved in DMSO to make a 10 mM stock solution and was added directly to the medium at different concentrations. CellTiter-Glo(^®^) luminescent cell viability assay was purchased from Promega (Madison, WI), and Transwell inserts and Matrigel were obtained from BD Biosciences. Cells were treated with 0.1% DMSO as the control group.

### Cell viability assay

Cells were seeded at 5 × 10^3^ cells/well in 96-well plates for 24 h and then treated with different concentrations of rottlerin. After 48 h and 72 h treatment, cell viability was assessed using the CellTiter-Glo^®^ luminescence (CTG) assay. Each value was normalized to cells treated with DMSO.

### Colony forming assay

MCF-7 and MDA-MB-231 cells were cultured in a 6-well plate at 1 × 10^3^ cells/well treated with different concentrations of rottlerin. Cells were grown for 15 days. Colonies were fixed by 4% paraformaldehyde and stained with crystal violet to enable enumeration of colonies.

### Cell apoptosis analysis

Cells (2 × 10^5^ cells/well) were cultured in a six-well plate overnight and treated with various concentrations of rottlerin for 48 h. Then, cells were harvested and washed with PBS, resuspended in 500 μl binding buffer with 5 μl Propidium iodide (PI) and 5 μl FITC-conjugated anti-Annexin V antibody. Apoptosis was analyzed by a FACScalibur flow cytometer (BD, USA).

### Cell cycle analysis

Exponentially growing cells (2.5 × 10^5^ cells/well) were seeded in a 6-well plate overnight and then treated with 3 μM and 5 μM rottlerin for 48 h. After 48 h, cells were collected and washed with cold PBS. Then, suspended cells with 70% cold alcohol were kept at 4°C overnight. Prior to analysis, the cells were washed with cold PBS, and re-suspended at 1 × 10^6^ cells/ml in PBS. Cells were incubated with 0.1 mg/ml RNase I and 50 mg/ml Propidium iodide (PI) for 30 min. Cell cycle was analyzed with a FACScalibur flow cytometer (BD, USA).

### Cell wound healing assays

MCF-7 and MDA-MB-231 cells were cultured in 6-well plates. After cells converged almost 100%, absorbed the supernatant and scratched the cells with a yellow pipette tip. Then washed the cells with PBS and added medium with rottlerin. The scratched area was photographed with a microscope at 0 h and 20 h, respectively.

### Cell invasion assay

Cell invasion assay was conducted to test the invasive activity of MCF-7 and MDA-MB-231 cells treated with rottlerin or Skp2 transfection or combination. Briefly, transfected cells were seeded in the upper chamber with 200 μl serum-free medium and there is 500 μl complete medium in the under chamber with the same concentration of rottlerin. After incubation for 20 h, the membrane of the chamber was strained with Giemsa and photographed with a microscope [40].

### Transfection

Cells were seeded into 6-well plates and transfected with Skp2 cDNA or Skp2 siRNA or empty vector using lipofectamine 2000 following the instruction's protocol [40]. Skp2 siRNA: sense 5′-GGAGUGACAAAGACUUUG UTT-3′; antisense 5′-ACAAAGUCUUUGUCACUC CTT-3′. After the transfection, the cells were subjected to further analysis as described under the results sections.

### Quantitative real-time reverse transcription-PCR analysis

The total RNA was extracted with Trizol (Invitrogen, Carlsbad, CA) and reversed-transcribed into cDNA by RevertAid First Strand cDNA Synthesis Kit. PCR were performed using Power SYBR Green PCR Master Mix and the results were calculated by 2-ΔΔCt method as described previously [40]. The primers used in the PCR reaction are: Skp2, forward primer (5′–GCTGCTAAAGGTCTCTGG GT-3′) and reverse primer (5′-AGGCTTAGATTCTGC AC TTG-3′); GAPDH, forward primer (5′-ACCCAGAAG ACTGTGGATGG-3′) and reverse primer (5′-CAGTGA GCTTCCCGTTCAG-3′).

### Western blotting analysis

The harvested cells were washed by PBS and lysed with protein lysis buffer. The concentrations of the proteins were tested by BCA Protein Assay kit (Thermo Scientific, MA). Same amount of protein samples were separated by electrophoresis in Sodium Dodecyl Sulfonate (SDS)-polyacrylamide gel and then transferred onto a Polyvinylidene Fluoride (PVDF) membrane, and then incubated with primary antibody at 4°C overnight. After washed with TBST for three times and incubated with second antibody at room temperature for one hour. Then the expression of protein was detected by electrochemiluminescence (ECL) assay [43].

### Statistical analysis

All statistical analyses were conducted using GraphPad Prism 4.0 (Graph Pad Software, La Jolla, CA). Student's *t*-test was performed to evaluate statistical significance. Results were presented as means ± SD. *P* < 0.05 was considered as statistically significant.

## SUPPLEMENTARY MATERIAL FIGURE


