# Supplementary material for: Rottlerin exerts its anti-tumor activity through inhibition of Skp2 in breast cancer cells
Source: Oncotarget. 2016 Aug 25;7(41):66512–24. doi: 10.18632/oncotarget.11614 (PMC5341817; doi:10.18632/oncotarget.11614)
Supplement: Supplementary file 1 [file oncotarget-07-66512-s001.pdf]

Rottlerin exerts its anti-tumor activity through inhibition of Skp2 in breast cancer cells

Supplementary Materials

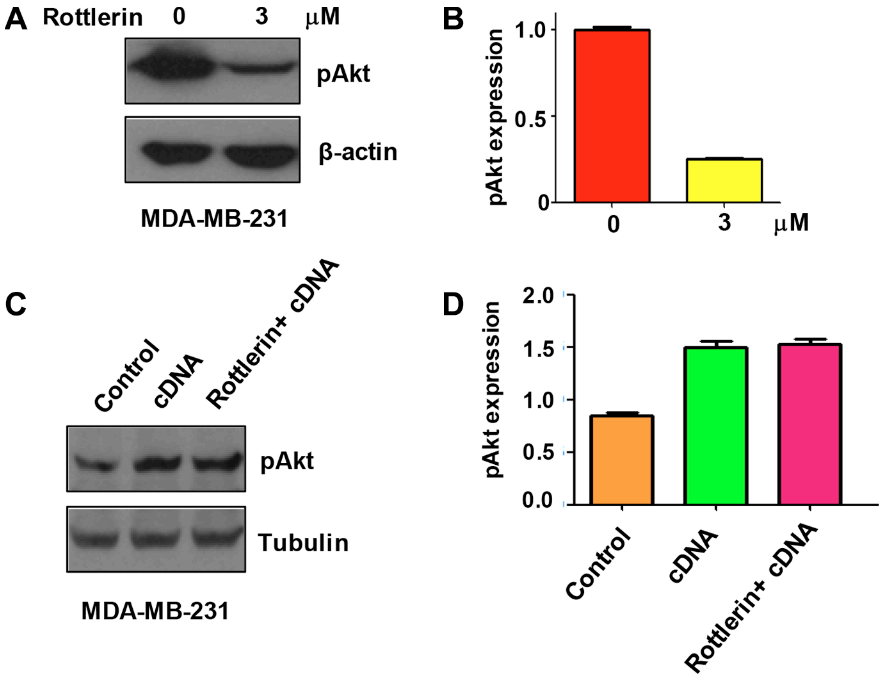

Supplementary Figure S1: Overexpression of Skp2 abrogated the inhibition of pAkt by rottlerin. (**A**) Western blotting analysis was conducted to determine the expression of pAkt in MDA-MB-231 cells after rottlerin treatment.(**B**) Quantitative results are illustrated for panel A. (**C**) The expression of pAkt was measured by Western blotting in MDA-MB-231cells with Skp2 cDNA transfection and rottlerin treatment. Control: pcDNA3.1; cDNA: Skp2 cDNA; Rottlerin+cDNA: rottlerin+Skp2 cDNA. (**D**) Quantitative results are illustrated for left panel C.
